# Assessment of the Effectiveness of Vitamin Supplement in Treating Eczema: A Systematic Review and Meta-Analysis

**DOI:** 10.1155/2019/6956034

**Published:** 2019-10-31

**Authors:** Ziyu Zhu, Ziyi Yang, Chunyi Wang, Handeng Liu

**Affiliations:** ^1^Laboratory of Tissue and Cell Biology, Experimental Teaching and Management Center, Chongqing Medical University, Chongqing 400016, China; ^2^The First Clinical College, Chongqing Medical University, Chongqing 400016, China; ^3^College of Pediatrics, Chongqing Medical University, Chongqing 400016, China

## Abstract

**Background:**

The morbidity of eczema has increased in the recent years, and the methods to prevent or ameliorate its effects are becoming more important. To this end, this research was conducted to determine the effectiveness of vitamin supplements in eczema therapy.

**Method:**

Embase, PubMed, and Cochrane Central Register of Clinical Trials were searched. Only randomized controlled trials were included, and we included all quantified eligible data where the SCORing Atopic Dermatitis (SCORAD) Index or Eczema Area and Severity Index (EASI) scores were applied to assess the severity of eczema.

**Results:**

Ten studies fulfilled the inclusion criteria, and eight of them were included for quantitative analysis (total: 456 patients). Compared to the controls, the SCORAD index or EASI decreased in the vitamin supplement group (mean difference −5.96, 95% CI: −7.69 to −4.23 for vitamin D3; mean difference −5.72, 95% CI: −11.41 to −0.03 for vitamin E; and mean difference −3.19, 95% CI: −4.27 to −2.10 for vitamin B12).

**Conclusion:**

This study suggests that vitamin supplements could be important therapeutics to help manage eczema patients.

## 1. Introduction

Eczema, a common skin irritation, often presents as a chronic, inflammatory, and pruritic skin abnormality. It encompasses a group of conditions, among which atopic dermatitis (AD) is the most prevalent type. Recently, the occurrence or prevalence of atopic dermatitis has increased in Africa, eastern Asia, and Western Europe [1]. Though drugs can effectively manage the symptoms and improve the life quality of most patients, a radical cure has yet to be found.

Vitamins play significant roles in maintaining normal body function. For instance, vitamin D, especially vitamin D3, had been linked to dermatological disorders, including atopic dermatitis. The metabolite of D3, 1*α*,25-dihydroxyvitamin D3 [2, 3], as well as vitamin E and vitamin C, have long been applied for skin care as antioxidants to maintain cellular redox balance [4]. Vitamin B12, also known as cobalamin, has a complex relationship with the skin and is thought to help lessen eczema symptoms by reducing nitric oxide levels [5–7]. Previous studies have suggested that vitamins, especially vitamin E, vitamin D, and vitamin B12, may be useful in the treatment of atopic eczema (AE); however, more evidence is needed before vitamin supplements can be recommended for atopic eczema [7]. For example, although the use of vitamin D3 was found to be inversely associated with the severity of AD, the results of other studies have been conflicting [8]. Therefore, the present study was performed to ascertain the efficacy of vitamin supplements for treating eczema. Other meta-analyses have demonstrated vitamin supplementation to be a viable treatment option for eczema, but all the studies were limited to a single type of vitamin. Consequently, the present study included all types of vitamins and only randomized controlled trials using the SCORing Atopic Dermatitis (SCORAD) Index or the Eczema Area and Severity Index (EASI) scores were included for qualitative and quantitative analyses to ensure the quality of our study.

The scoring atopic dermatitis index is an evaluation system based on simplicity and ease of routine use in outpatient clinics. It evaluates the extent, severity, and subjective symptoms (edema/papulation, erythema excoriation, oozing/crust, lichenification, and dryness) of AD [9]. EASI is an effective, easily understood tool for the evaluation of AD, focusing on crucial acute and chronic signs of inflammation (erythema, excoriation, induration/papulation, and lichenification) [10]. Quantitative analyses for vitamin D3, vitamin B12, and vitamin E were conducted in the present study.

## 2. Materials and Methods

This meta-analysis was performed in accordance with the Cochrane Handbook for Systematic Reviews of Interventions and followed the Preferred Reporting Items for Systematic Reviews and Meta-Analyses (PRISMA) rules. The protocol for this meta-analysis was registered on PROSPERO (CRD42018117330).

### 2.1. Search Strategy and Data Collection

Electronic databases, including PubMed (up to January 2019), Embase (up to January 2019), and Cochrane Central Register of Clinical Trials (up to January 2019), were searched using the keywords “eczema AND vitamin” to identify studies on patients diagnosed with eczema using clinical diagnoses or validated diagnostic criteria. The detailed search strategy can be accessed in Additional File 1.

**Figure 1:**
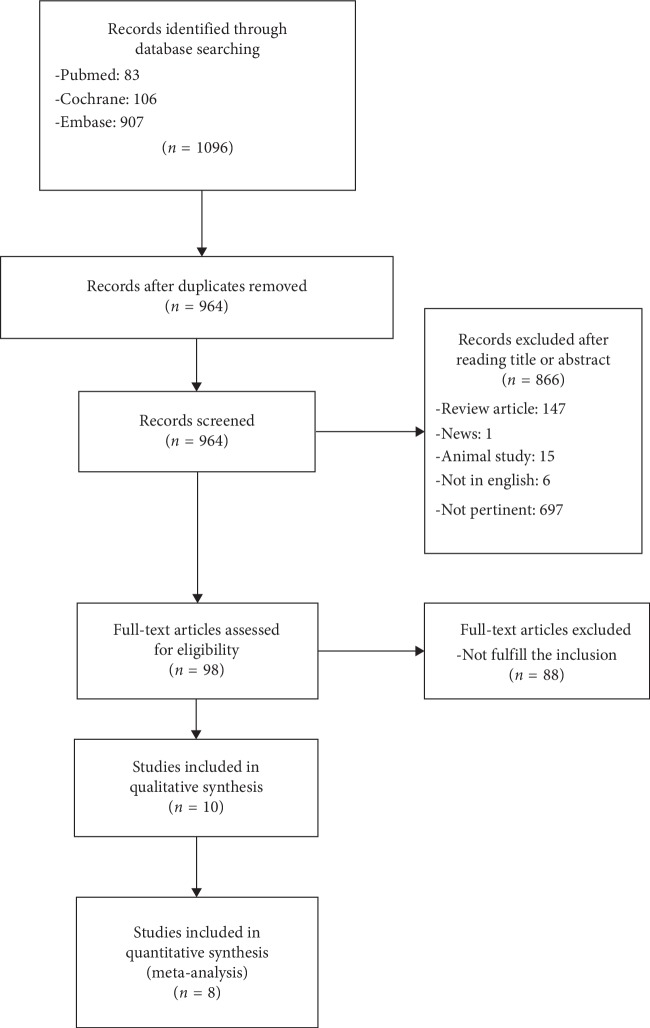
Flow diagram of the study screening process.

All the search results were screened by two independent reviewers. Initially, the studies were identified by titles, and then the abstracts of the related papers were assessed to find articles for further full-text screening before final inclusion in the systematic review. A flow diagram of the selection process is shown in Figure 1.

### 2.2. Study Selection and Data Extraction

Studies met the following criteria for inclusion in our analyses: (1) participants: eczema patients with a clinical diagnosis; (2) interventions: information on vitamin supplements, dosage intervals, dosage, and route of administration. The types of vitamins were not restricted. The comparator was limited; only studies which examined placebo groups or no vitamin supplement groups were included; (3) outcome measures: the SCORAD index or EASI; and (4) types of studies: only articles on randomized controlled trials (RCT) written in English were included. Animal studies, case reports, and review articles were omitted.

All data were obtained in duplicate from the included articles and were recorded on a form designed independently by two of the authors in advance. No blending of authorship existed in the process. Data extracted from each article included authors, countries, publication year, study design, inclusion and exclusion criteria, interventions introduced, number randomized and analyzed, and outcomes measured by the SCORAD Index or EASI scores.

### 2.3. Quality of Assessment

**Figure 2:**
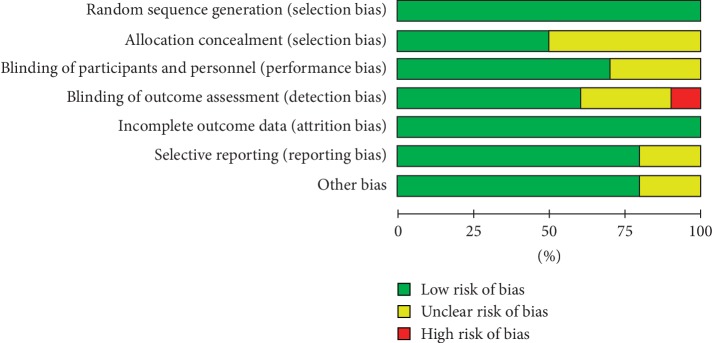
Risk of bias graph of all included studies.

**Figure 3:**
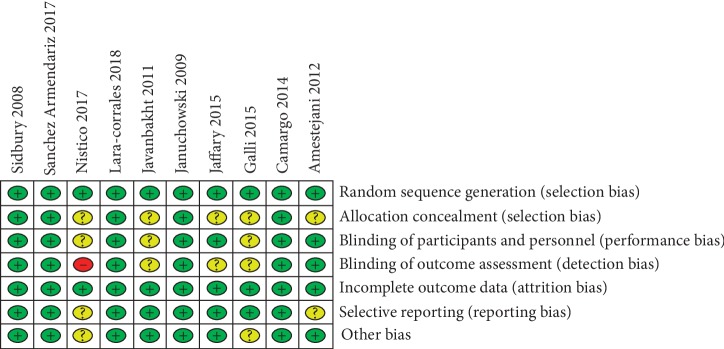
Risk of bias summary for each included study.

**Figure 4:**
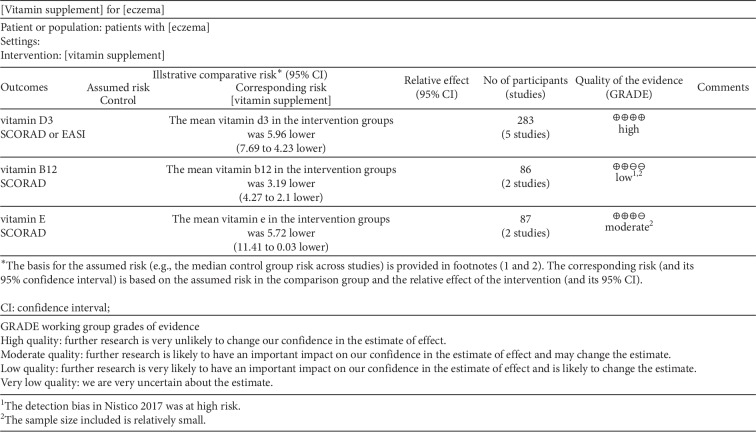
Summary of findings.

**Figure 5:**
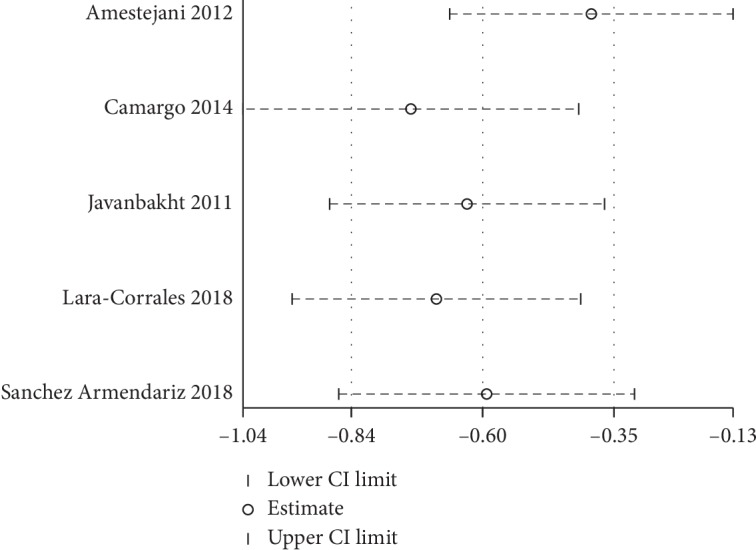
Sensitivity analysis for included studies on vitamin D3 supplementation.

Discrepancies between the two reviewers were resolved by discussion or by obtaining the opinion of a third evaluator, and a consensus was finally achieved. The assessment of bias was conducted using the Cochrane Collaboration Risk of Bias Tool for randomized controlled trials. The risks assessed included random sequence generation, allocation concealment, incomplete outcome data, blinding, selective reporting, and other biases. After individual rating and discussion, a consensus was reached (Figures 2 and 3). To rate the quality of the evidence, the Grading of Recommendations, Assessment, Development, and Evaluation (GRADE) was used, according to the five downgraded criteria: risk of bias, inconsistency, indirectness, imprecision, and publication bias (Figure 4). In addition, a sensitivity analysis was performed to test every single study's influence on the stability of the entire quantitative analysis for vitamin D3 (Figure 5).

### 2.4. Statistical Analyses

Fixed effects models were used to calculate the weighted mean difference or standardized mean difference (MD) with 95% confidence intervals (CI) to assess the effectiveness of vitamin use to relieve or prevent eczema using Cochrane Collaboration's software (RevMan 5.3). A sensitivity or specificity test was conducted using STATA 15.1. The correlation factor suggested by the Cochrane Collaboration Tool was set to 0.5 to calculate variance. Heterogeneity was checked using *I*^2^ statistics, and meaningful heterogeneity was determined by 50% of the *I*^2^ value.

## 3. Results

The initial search identified 1096 studies, and 98 were selected for full-text screening. Only 10 studies evaluated the SCORAD Index or EASI score in eczema patients and controls, fulfilling the inclusion criteria. They were included for qualitative analysis, and eight of them were included in the quantitative synthesis. All the included articles assessed the effectiveness of vitamin supplements in eczema patients using the SCORAD Index or EASI scores. The characteristics of the included articles are summarized in Table 1.

### 3.1. Comparison of the SCORAD Index or EASI Scores of the Vitamin Supplement Group and Placebo Group

Ten RCTs were included in the review. The sample sizes ranged from 5 to 57. The studies were published from 2008 to 2018. Five studies were conducted on atopic dermatitis, three studies were conducted on pediatric atopic dermatitis, and two studies were conducted on winter-related atopic dermatitis.

Compared to the control group, the SCORAD Index of the patients given different vitamins was significantly lower, especially in the study by Javanbakht et al. [12], where combined supplementation with both vitamin E and vitamin D3 was better than any other single vitamin used in that study (−23.1 for combined vitamins group vs. −12.9 for single vitamin E group).

### 3.2. Vitamin D3 Supplementation in Atopic Dermatitis Patients

In all, the data from 148 atopic dermatitis patients and 135 controls in the RCTs were included in the quantitative analysis. Amestejani et al. [11] only included patients aged 14 or older, Javanbakht et al. [12] included patients aged 13 to 45 years old, and Lara-Corrales et al. [14] included patients between the ages of 0 and 18 years. Armendariz et al. [13] included patients aged 2–54, and Camargo et al. [18] included patients with a mean age of 9 (SD = 5).

**Figure 6:**
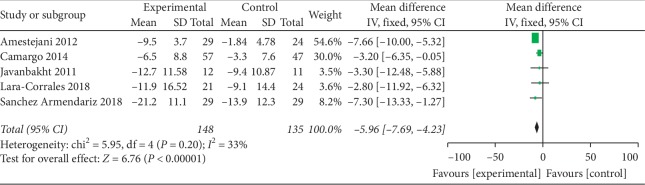
Forest plot of the meta-analysis of vitamin D3 and atopic dermatitis.

The results showed that the SCORAD Index or EASI scores decreased in patients given vitamin D3 (cholecalciferol) supplements (mean difference −5.96, 95% CI: −7.69 to −4.23). The *I*^2^ statistic suggested that no meaningful heterogeneity was detected across the studies (*I*^2^ = 33%) (Figure 6).

### 3.3. Vitamin B12 Supplementation in Atopic Dermatitis Patients

**Figure 7:**
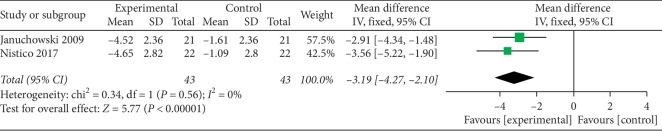
Forest plot of the meta-analysis of vitamin B12 and atopic dermatitis.

Two randomized controlled trials testing the effectiveness of vitamin B12, both measured by the SCORAD index, were included in our analysis. The results showed that the SCORAD Index decreased after the topical application of vitamin B12-containing cream (mean difference −3.19, 95% CI: −4.27 to −2.10). No meaningful heterogeneity was detected (*I*^2^ = 0%) (Figure 7).

### 3.4. Vitamin E Supplementation in Atopic Dermatitis Patients

**Figure 8:**
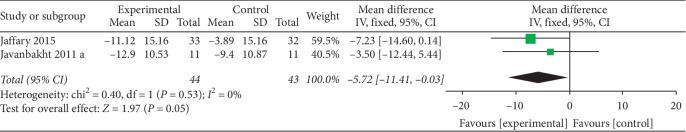
Forest plot of the meta-analysis of vitamin E and atopic dermatitis.

Two randomized controlled trails testing vitamin E on patients with eczema, both measured by the SCORAD Index, were included in our analysis. The results suggested that the SCORAD Index improved after vitamin E supplementation (mean difference −5.72, 95% CI: −11.41 to −0.03). No meaningful heterogeneity was detected (*I*^2^ = 0%) (Figure 8).

### 3.5. Adverse Effects

Javanbakht et al. [12] reported two exacerbated patients: one in the placebo group (11.7%) and one in the vitamin E group (27.7%). The study by Januchowski [15] reported that one patient withdrew earlier due to apparent side effects of both the placebo and study creams.

## 4. Discussion

In the present study, we included and comprehensively analyzed randomized controlled trials and generated potentially important conclusions. According to the results of the quantitative analyses in the RCTs (Amestejani et al. [11], Javanbakht et al. [12], Lara-Corrales et al. [14], and Armendariz et al. [13]), the condition of atopic dermatitis patients, particularly those with moderate-to-severe disease, was improved after treatment with vitamin D3 (cholecalciferol) supplements (mean difference −7.15, 95% CI: −9.21 to −5.08). Additionally, Jaffary et al. [16] and Javanbakht et al. [12] administrated 400 IU and 600 IU of vitamin E, respectively, and showed favorable improvement in the SCORAD Index (SCORAD change: −11.12 compared to the placebo group: −3.89, *p* < 0.05; SCORAD change: −12.9 compared to placebo group: −9.4, respectively). Of note, the studies by Januchowski et al. [15] and Nistico et al. [20] treated patients with a topical cream containing vitamin B12 (0.07% cyanocobalamin by weight in a moisturizing base and 0.07% vitamin B12 in a barrier cream embedded in a polysorbate carrier system designed to penetrate the skin with a lipid concentration of 24% and 1% urea, respectively). Their results suggested improvements as well (SCORAD change = -4.52 compared with placebo = −1.61 by Januchowski et al. [15] and SCORAD change = −4.65 compared with placebo = −1.09 by Nistico et al. [20]). In the study by Javanbakht et al. [12], the researchers designed a vitamin E and vitamin D3 combined group, as well as single-supplemented groups to assess the effect of the treatments on the clinical manifestation of atopic dermatitis (SCORAD change: −23.1 compared with placebo: −9.4).

Vitamin D is in a family of nutrients that share similarities in chemical structures. Vitamin D2 (ergocalciferol) and vitamin D3 (cholecalciferol) are the most common members of this large family. General speaking, vitamin D3 is more effective in improving vitamin D status, though both are important in maintaining health [21, 22]. Hence, the study by Sidbury et al. [17] was not consolidated with the other studies using vitamin D3 supplements because vitamin D2 was evaluated in that trial. With the aim of assessing the durability of the response to treatment with vitamin supplements, only Jaffary et al. [16] analyzed relapse rates. Twenty-five percent of the patients in the treatment group (7/28) versus 22.2% in the placebo group (6/21) relapsed after treatment with vitamins.

Presently, through a set of systematic remedies ranging from emollients to corticosteroids, we are able to manage most of the symptoms of eczema [23]. Although the exact etiology of eczema is still unknown, a combination of genetic and environmental factors is involved. Dysfunction of the natural skin barrier has been considered one of the main pathophysiologic theories. It occurs not as a driver but as a consequence of eczema, and filaggrin deficiency has been implicated in the pathogenesis of atopic dermatitis due to its significant role in homeostasis of the skin [24, 25]. The patient's immune function and dysfunction are involved in the pathogenesis, too, which involves a sophisticated network of immune response proteins [26]. For instance, from deeply-sequenced RNA samples using long paired-end reads, a recent study reported that atopic dermatitis was an IL-13-dominated disease [27]. In another study, the researchers suggested that inflammasome-dependent IL-1β was involved in the pathogenesis based on a confirmed correlation between patients' AD severity and IL-1Ra [28]. A systematic review assessed 48 publications and also suggested that IL-17, as well as IL-22, seemed to play roles in the pathogenesis of allergic skin maladies [29]. Further studies are needed to accurately identify the mechanism of eczema progression. Meanwhile, connections between vitamins and the disease are being increasingly discovered. A recent study reported that vitamin D3 might ameliorate atopic dermatitis by producing tolerogenic dendritic cells to the allergic phenotype in children [30]. Vitamin E may affect the immune function directly by altering the membrane function or by indirectly affecting a sophisticated signaling pathway by affecting inflammatory mediators [31]. Vitamin C was found to improve the overall epidermal barrier function by modulating ceramide metabolic-related enzymes, thereby increasing ceramide production [32]. Nevertheless, to fully elucidate the specific mechanisms involved, far more studies are needed.

Our results agree with other similar systematic reviews and meta-analyses [33–35], which determined the efficacy of vitamin D supplements to treat atopic dermatitis. However, this study only included randomized controlled trials measuring outcomes with the SCORAD Index or EASI scores, and the types of vitamins analyzed were not limited to a single one. Of note, in a study conducted by Galli et al. [19], 3-month consecutive supplementation with vitamin D3 (2000 IU daily) failed to show a statistical correlation between the serum levels of vitamin D and eczema severity [36]. In comparison with the other five studies, the mean baseline of the SCORAD index in that study was much lower (12.2 in Galli et al. [19], 24.8 in Amestejani et al. [11], 36 in Javanbakht et al. [12], 27.3 in Lara-Corrales et al. [14], and 41.3 in Armendariz et al. [13]). The severity of eczema in this study was evaluated according to the commonly used SCORAD classification (mild for 0–25, moderate for 26–50, and severe for 51–103), which seems to be an update from the experts of the previously suggested severity strata for oSCORAD in 1997 [30, 31]. Therefore, we suspect that the lack of outcome correlations might be explained by the comparison of different interpretations of the SCORAD Index and total baseline scores. After a detailed review of the five other studies analyzing the baseline scores and SCORAD cutoffs in each study, we found that Galli et al.'s study [19] was the only study whose enrolled experimental group could be considered as having mild eczema, while patients in the other five studies had moderate eczema. In the study by Camargo et al. [18], 89% of the 107 children had moderate disease. Moreover, in recent studies, researchers found that the serum vitamin D levels in patients with milder forms of atopic dermatitis were significantly higher, which might help clarify the disparate outcomes [37–40].

In conclusion, the present study summarized the latest evidence for treating atopic dermatitis patients by administrating vitamin supplements. Due to the limited number of included studies, meta-analysis was conducted for several kinds of vitamins used in the selected studies. It confirmed the favorable efficacy of this daily treatment, especially in patients with moderate-to-severe AD. In addition, vitamin E and vitamin B12 were also found beneficial for AD patients. Our conclusions should be interpreted with some caveats. First, the total sample size was still relatively small. Second, potential confounding factors, such as the use of topical steroids or calcineurin inhibitors, were not adjusted. Despite these limitations, we still consider this study useful to verify the positive effects of vitamin supplements to relieve the severity of patients' symptoms. Larger randomized controlled trials are required to determine the optimum ways to use vitamin supplements to treat atopic dermatitis, and thus, manage patients with different severities using the most effective supplement possible.

## Figures and Tables

**Table 1:** Characteristics of the included studies.

First author year (Reference)	Country	Study design	Sample size (I vs. C)	Inclusion criteria	Exclusion criteria	Intervention	Control	Main outcome	Mean change (I vs. C)
Amestejani 2012 [11]	Iran	RCT	29 vs. 24	Age 14 and older, with no concomitant pyretic, systemic disease or inflammatory processes^1^	Pregnancy, concomitant systemic inflammatory disorder^1^, and use of vitamins, minerals and fatty acids supplements, oral contraceptive pills, antiepileptic agents, steroid hormones^2^, anticoagulant drugs	1600 IU vitamin D3 60 days	Placebo filled with starch identical in size and color	SCORAD	−9.5, −1.84
Javanbakht 2011 [12]	Iran	RCT	GD^3^: 12 GE^4^: 11 GDE^5^: 11 vs. GP^6^: 11	Patients with SCORAD from 10 to 70	Patients taking vitamins, minerals and fatty acids supplements, oral contraceptive pills, steroid hormones^2^, antiepileptic agents, anticoagulant drugs, and pregnant or nursing mothers	GD^3^: 1600 IU vitamin D3 and vitamin E placebo daily; GE^4^: 600 IU vitamin E and vitamin D3 placebo daily; GDE^5^: 1600 IU vitamin D3 and 600 IU vitamin E placebo daily 60 days	Vitamin D3 placebo filled with starch, vitamin E placebo filled with mineral oil	SCORAD	GD^3^: −12.7, −9.4 GE^4^: −12.9, −9.4 GDE^5^: −23.1, −9.4
Sanchez Armendariz 2018 [13]	Mexico	RCT	29 vs. 29	Patients diagnosed with moderate-severe AD	Patients with primary immunodeficiency, renal tubular acidosis, and pregnancy and those taking other supplements, as well as lack of follow-up at 12 weeks	5000 IU vitamin D3 daily for 3 months	Cellulose	SCORAD	−21.2, −13.9
Lara-Corrales 2018 [14]	Canada	RCT	21 vs. 24	Patients aged between 0 and 18 with a clinical diagnosis of AD	Patients with known renal or liver disease or chronic dermatological conditions other than AD	2000 IU vitamin D3 (2 drops of vitamin D3, 1000 IU/drop) daily for 3 months	Identical-appearing placebo drops	SCORAD	−15.4, −15.3
Januchowski 2009 [15]	America	RCT	21 vs. 21	Children aged 6 months to 18 years with eczema and the ability to understand the consent process	Unwillingness of the parents to consent to the study protocol; pregnancy or lactation; eczema with superinfection present; known history of allergy to vitamin B12 or components of the base cream; topical treatment with corticosteroids in the 4 weeks prior to enrollment; or inability to speak and read English	0.07% vitamin B12 twice a day for 4 weeks	The same moisturizing base without vitamin B12	SCORAD	−4.52, −1.61
Jaffary 2015 [16]	Iran	RCT	33 vs. 32	Patients aged 10–50 years with a clinical diagnosis of AD	Severe disease, requiring hospitalization, pregnant women, nursing mothers, coagulopathies, use of anticoagulant medications, systemic corticosteroids or immunosuppressants, as well as having a history of allergy to vitamin E, and living in long distances from Isfahan city; those patients who showed severe allergic symptoms to vitamin E and those who became pregnant during the therapy	400 IU vitamin E daily 4 months oral	Placebo with no active ingredient and without smell	SCORAD	−11.12, −3.89
Sidbury 2008 [17]	America	RCT	5 vs. 6	Unclear	Unclear	1000 IU vitamin D2 daily for 1 month	Identical looking placebo	EASI	−4.6, −2.2
Camargo 2014 [18]	Mongolia	RCT	57 vs. 47	Patients aged 2–17 years with winter-related AD	Patients with an active skin infection	1000 IU vitamin D3 daily for 1 month	Colorless, odorless and tasteless placebo	EASI	−6.5, −3.3
Galli 2015 [19]	Italy	RCT	41 vs. 48	Children with chronic eczema (48 boys) with a median age of 68 months (range 6–195 months), with a clinical diagnosis of AD	Unclear	2000 IU vitamin D3 daily for 3 months	No vitamin D3 supplementation	SCORAD	−0.2, −1.3
Nistico 2017 [20]	Italy	RCT	22 vs. 22	Male or female Caucasian patients with a confirmed clinical diagnosis of mild AD measured with total SCORAD Index up to 25 points	Unclear	0.07% vitamin B12 imbedded in a polysorbate carrier system 3 times a day for 12 weeks	Glycerol-petrolatum-based emollient cream	SCORAD	−4.65, −1.09

^1^Other than diabetic mellitus and chronic viral hepatitis. ^2^Oral or parenteral. ^3^Vitamin D3 supplement group. ^4^Vitamin E supplement group. ^5^Vitamin D3 and vitamin E supplements in combined group. ^6^Placebo group.

## Data Availability

The data used to support the findings of this study are available from the corresponding author upon request.

## Supplementary Materials

Supplementary Materials Table S1: detailed search strategy and results supporting this study (Embase, PubMed and the Cochrane Central Register of Clinical Trails). All the search history was retrieved from the database website. Table S2: the raw data extracted and processed for further quantitative analysis of vitamin supplement effectiveness.
