# Supplementary material for: Assessment of the Effectiveness of Vitamin Supplement in Treating Eczema: A Systematic Review and Meta-Analysis
Source: Evid Based Complement Alternat Med. 2019 Oct 31;2019:6956034. doi: 10.1155/2019/6956034 (PMC6875217; doi:10.1155/2019/6956034)
Supplement: Supplementary Materials — Table S1: detailed search strategy and results supporting this study (Embase, PubMed and the Cochrane Central Register of Clinical Trails). All the search history was retrieved from the database website. Table S2: the raw data extracted and processed for further quantitative analysis of vitamin supplement effectiveness. [file 6956034.f1.zip › Table S1.pdf]

Pubmed

#15	Add	Search **(((randomized controlled trial[Publication Type] OR randomized[Title/Abstract] OR placebo[Title/Abstract]))) AND (((("Vitamins"[Mesh]) OR Vitamin[Title/Abstract])) AND ((eczema) OR ((((((Dermatitis[Title/Abstract]) OR Eczematous[Title/Abstract]) OR Dermatitides[Title/Abstract]) OR Eczematous[Title/Abstract]) OR Eczematous Dermatitides[Title/Abstract]) OR Eczematous Dermatitis[Title/Abstract])))**	83	03:57:17
#14	Add	Search **(randomized controlled trial[Publication Type] OR randomized[Title/Abstract] OR placebo[Title/Abstract])**	767521	03:57:00
#13	Add	Search **((("Vitamins"[Mesh]) OR Vitamin[Title/Abstract])) AND ((eczema) OR ((((((Dermatitis[Title/Abstract]) OR Eczematous[Title/Abstract]) OR Dermatitides[Title/Abstract]) OR Eczematous[Title/Abstract]) OR Eczematous Dermatitides[Title/Abstract]) OR Eczematous Dermatitis[Title/Abstract]))**	898	03:56:25
#12	Add	Search **("Vitamins"[Mesh]) OR Vitamin[Title/Abstract]**	203398	03:55:36
#11	Add	Search **(eczema) OR ((((((Dermatitis[Title/Abstract]) OR Eczematous[Title/Abstract]) OR Dermatitides[Title/Abstract]) OR Eczematous[Title/Abstract]) OR Eczematous Dermatitides[Title/Abstract]) OR Eczematous Dermatitis[Title/Abstract])**	71319	03:55:22
#10	Add	Search **Vitamin[Title/Abstract]**	187137	03:55:03
#9	Add	Search **(((((Dermatitis[Title/Abstract]) OR Eczematous[Title/Abstract]) OR Dermatitides[Title/Abstract]) OR Eczematous[Title/Abstract]) OR Eczematous Dermatitides[Title/Abstract]) OR Eczematous Dermatitis[Title/Abstract]**	57091	03:52:48
#6	Add	Search **"Eczema"[Mesh]**	10723	03:47:48
#5	Add	Search **"Vitamins"[Mesh]**	36246	03:46:32
#1	Add	Search **eczema**	20290	03:39:20

Embase

Session Results

....

No.	Query Results	Results	Date
#14.	#12 AND #13	907	19 Jan 2019
#13.	'clinical trial'/de OR 'randomized controlled trial'/de OR 'randomization'/de OR 'single blind procedure'/de OR 'double blind procedure'/de OR 'crossover procedure'/de OR 'placebo'/de OR 'prospective study'/de OR ('randomi?ed controlled' NEXT/1 trial*) OR rct OR 'randomly allocated' OR 'allocated randomly' OR 'random allocation' OR (allocated NEAR/2 random) OR (single NEXT/1 blind*) OR (double NEXT/1 blind*) OR ((treble OR triple) NEAR/1 blind*) OR placebo*	2,087,802	19 Jan 2019
#12.	#10 AND #11	4,414	19 Jan 2019
#11.	#1 OR #3 OR #4 OR #5 OR #6	97,609	19 Jan 2019
#10.	#2 OR #9	642,407	19 Jan 2019
#9.	vitamins:ab,ti	35,927	19 Jan 2019
#8.	eczematous AND dermatitis:ab,ti	2,094	19 Jan 2019
#7.	eczematous AND dermatitides:ab,ti	12	19 Jan 2019
#6.	eczematous:ab,ti	3,551	19 Jan 2019
#5.	dermatitides:ab,ti	188	19 Jan 2019
#4.	eczematous:ab,ti	3,551	19 Jan 2019
#3.	dermatitis:ab,ti	74,499	19 Jan 2019
#2.	'vitamin'/exp	634,500	19 Jan 2019
#1.	'eczema'/exp	28,463	19 Jan 2019

....

Cochrane

Search Name:
Date Run:20/01/2019 07:49:36
Comment:

ID	Search	Hits
#1	MeSH descriptor: [Eczema] explode all trees	856
#2	MeSH descriptor: [Vitamins] explode all trees	3693
#3	(Dermatitis):ti,ab,kw (Word variations have been searched)	6058
#4	(Eczematous):ti,ab,kw (Word variations have been searched)	167
#5	(Dermatitides):ti,ab,kw (Word variations have been searched)	9
#6	(Eczematous):ti,ab,kw (Word variations have been searched)	167
#7	(Eczematous Dermatitides):ti,ab,kw (Word variations have been searched)	1
#8	(Eczematous Dermatitis):ti,ab,kw (Word variations have been searched)	127
#9	(Vitamin):ti,ab,kw (Word variations have been searched)	22438
#10	#1 OR #3 OR #4 OR #5 OR #6 OR #7 OR #8	6396
#11	#2 OR #9	22438
#12	#10 AND #11	106
